# Solidarity against healthcare access restrictions on undocumented immigrants in Spain: the REDER case study

**DOI:** 10.1186/s12939-019-0971-9

**Published:** 2019-06-06

**Authors:** Maider Urtaran-Laresgoiti, Janire Fonseca Peso, Roberto Nuño-Solinís

**Affiliations:** 1 Deusto Business School Health, University of Deusto, Hermanos Aguirre, 2 -3ª planta, 48014 Bilbao, Spain; 2 Facultad de Psicología y Educación, Departamento de Pedagogía Social y Diversidad, University of Deusto, Bilbao, Spain

## Abstract

**Background:**

In the context of public expenditure reduction and cuts, in 2012, the Spanish government approved the RDL 16/2012, which significantly affected the core values of the national health system. The measure particularly affected undocumented immigrants over 18 years of age, excluding them from accessing the full range of healthcare services in Spain, except for emergency care. In 2014, Red de Denuncia y Resistencia al RDL 16/2012 (REDER) was created as a public awareness and resistance network to defend universal access to healthcare and to stop its infringement. This study aims to analyse the social impact of REDER as a solidarity movement in response to the exclusion of undocumented immigrants from their universal right to health.

**Methods:**

Qualitative research methodologies were used for the research. Data were collected between November 2017 and December 2017, using eight semi-structured interviews with key informants from the main REDER stakeholders. Additionally, key publications, documents, and presentations of researchers and experts in the field were analysed. For data analysis, a framework extracted from the literature on exclusionary and transformative dimensions of solidarity was used to identify barriers and drivers in REDER’s intervention.

**Results:**

From its creation to the present, REDER has been able to achieve many of its objectives to defend the right to medical care of groups in irregular situations, contributing to the identification of 4,755 cases of discrimination in healthcare access and helping solve over 90% of these cases by delivering either healthcare assistance or administrative support. REDER has also played an important role in: stimulating social activation and empowering citizens to claim their fundamental rights, organising actions against restrictions on accessibility and creating synergies to restore universal healthcare coverage.

**Conclusions:**

REDER has been shown to be effective in leading the defence of universal healthcare rights, and some achievements in the years following 2012 could be directly attributed to the work done by the network, such as the elimination of legal requirements to obtain health cards or the reduction of the minimum time required to access healthcare. Despite context particularities, the initiatives and main actions of this network may be implemented in other settings that are facing similar limitations to healthcare access, in order to address injustices and promote solidarity.

**Electronic supplementary material:**

The online version of this article (10.1186/s12939-019-0971-9) contains supplementary material, which is available to authorized users.

## Background

The Spanish National Health System is considered a universal health system, funded by taxes and based on the principles of universality, equity, solidarity, quality and participation. However, in response to the huge impact of the economic and financial crisis in Spain, austerity measures agreed upon in the EU Stability Programme for the Kingdom of Spain resulted in a reduced public share of health expenditure, from 6.5% of the GDP in 2010 to 5.1% in 2015 [1–3].

Moreover, new legislation was issued to regulate coverage and access to healthcare, as well as benefits packages and the financial participation of patients, in the Spanish National Health System funding [3]. The enactment of the Royal Decree-Law 16/2012 (hereinafter RDL) and the subsequent Royal Decree 1192/2013 [4] challenged the principle of a universal healthcare system. In fact, until 2012, the Spanish National Health System coverage was almost universal (99.5%). However, the new RDL changed the basis for entitlement rights, linking the rights to the legal and working status of individuals. This change predominantly affected undocumented immigrants.

Although the reform affected the entire population, in practice, undocumented immigrants ended up excluded from the coverage and were only entitled to emergency care for serious illnesses or accidents until discharged, as well as to obstetric and child care [3]. Therefore, after implementation of the RDL in September 2012, approximately 873,000 non-residents lost entitlement to comprehensive care, including approximately 500,000 undocumented immigrants [5, 6].

Even if scientific evidence shows that ensuring broad primary care coverage and strengthening prevention and early detection are more efficient than providing care through emergency services [7, 8], the central government enacted the RDL for economic reasons and justified the urgency of the structural reform of the health system on its unsustainability. However, years later, a rigorous impact evaluation on cost containment or savings has not been conducted [9]. In contrast, the mortality rate in the immigrant community with irregular status increased by 15% over a three-year period, and there was a reduction in primary care utilisation and a greater use of emergency services in maternal and child care [10]. Furthermore, this law was adopted without considering the opinion of healthcare professionals or social organisations.

In 2014, Red de Denuncia y Resistencia al RDL 16/2012 –The Network for Reporting and Resisting the Royal Decree - Law 16/2012 (REDER) was created with the commitment to defend universal access to healthcare and to stop the infringement of access [11].

The REDER network was created as a citizen and social movement, and it is considered an expression of solidarity in the field of healthcare during crisis. Therefore, the analysis of its impact in terms of healthcare access, social cohesion and human rights protection can provide useful lessons for other countries facing similar challenges.

The present study is part of a European research project on solidarity expressions in European societies, called SOLIDUS. This project understands “solidarity” as a continuum of identity and contingency, as well as altruism and vulnerability. It is a concept that defies a singular definition, as the interpretation alternates depending on the context in which it is used, and the discipline within which it is being discussed (philosophy, political theory, sociology or psychology) [12]. The project aims to analyse in depth the acts of solidarity that are being developed across Europe, the extent to which they respond to dialogic and inclusive processes, and the related outcomes and policy developments. In this case, we focus the attention on the case of REDER.

Therefore, the aim of this study is to describe and analyse the social impact of REDER as a solidarity movement of professionals and citizens in response to restrictions on healthcare access.

## Methods

This research intends to understand the complex relations between solidarity (the main theme) and REDER (the case). Specifically, it intends to detect and examine the personal experiences of the people involved in REDER, which allows for the identification of detailed themes and opinions regarding solidarity [13]. To do so, a case study is used with a qualitative research methodology. Aimed to be exploratory in nature, data collection was carried out using two main techniques, i.e., pre-tested and semi-structured interviews and a content analysis of documents and information taken from a public meeting with experts in the field of public health and members of NGOs. The data analysis was carried out using previously established dimensions of solidarity from the SOLIDUS project.

This paper describes data related to the type of stakeholders involved in the organisation, what the stakeholders´ personal characteristics are, who develops the initiative, how decision-making is organised, what the organisation’s main purpose is, what type of organisation it is (public, private or mixed), what kind of logistics facilities are being used or provided by the organisation; and what kind of impact evidence is being observed. The research also includes a section that aims to describe how the analysed dimensions related to solidarity have played a key role in achieving its objectives.

### Key informant interviews

Key informants from principal stakeholders linked to REDER were interviewed during November and December of 2017. The aim of the selection criteria was to guarantee the representativeness and completeness of different perspectives. The participants were selected according to their

- knowledge or membership of the REDER movement;
- professional profile, i.e., public health, health policy, academic or third sector; and
- gender.

The key informants (*n* = 8) represented diverse profiles coming from civil society organisations (*n* = 5), policy makers (*n* = 1), and scholars (*n* = 2) (see Table 1). First, interviewees were selected according to their knowledge of or membership in REDER. A second criterion of inclusion was gender balance. The interviews were recorded with an electronic voice recorder and were conducted in Spanish by two research team members. Each interview took approximately 60 min.

**Table 1:** Interviewees, profiles and gender

Profile	Interviewee	Gender
Civil Society Organizations (CSO)	CSO1	M
	CSO2	F
	CSO3	M
	CSO4	F
	CSO5	F
Academia (A)	ACA1	M
	ACA2	F
Policy maker (PM)	PM1	M

Source: Elaborated by the authors

The guide for the interviews was based on the SOLIDUS project guidelines. The questions covered the actors´ understanding of how solidarity is organised, what the barriers are and how they are overcome, as well as the social and political impact deriving from them (see Additional file 1).

Transcription and translations (from Spanish to English) were performed and then transferred to word processing software (Microsoft Word). The transcripts were coded through ATLAS-ti.7 software, and emergent themes were identified from the codes. Before the interview, informed consent was obtained from all the participants after explaining the objectives of the study and their contribution. Participation was voluntary, and interviewees had the option to refuse to answer any question or withdraw at any time. During transcription, the respondents´ names were removed, and confidentiality was maintained throughout the study.

### Literature review and other sources of information

The review included published and unpublished reports (facilitated by the interviewees) such as annual reports, press releases, campaign reports, social media messages, videos, and other documents provided from the participating organisation, with the most up-to-date and accurate data. The extracted information contributed to the complete information from interviews.

The analysis of the documents was conducted using three key categories:

- characteristics of the organisation, the program or actions;
- impact on improving undocumented migrants´ standard of living (both in terms of healthcare provision and accessibility and social living); and
- measures at the policy level to respond to restrictions on healthcare in different autonomous communities and regional governments.

Furthermore, the policy seminar held as part of the National Public Seminars under the auspices of the European Research Project SOLIDUS in Madrid on April 2018, was used to gain a deeper insight into REDER. The event hosted a group of stakeholders involved in the REDER network, as well as people from academia who had conducted research regarding inequalities in healthcare access after approval of the RDL in Spain. Open in its nature, the seminar was organised with the aim of making interested audience aware of the achievements of REDER after its creation.

### Data analysis

Descriptive methods and descriptive codes were used for interpretation of the data extracted from the interviews. These methodologies set forth the elementary information regarding the reality with stronger methodological rigour and according to criteria established by the researchers [14].

According to Goetz and LeCompte [15], the strategies employed in reality provide phenomenological data, in that it represents the conception of the world of the participants who are being researched and from which the constructs that are going to structure the research are derived.

To examine the different characteristics of REDER in relation to data analysis, we used the SOLIDUS project analysis system; that is, the exclusionary (identifying barriers) and transformative (identifying drivers) dimensions of solidarity.

To do so, some key categories from the literature on solidarity were identified: Democracy [16–20], Pluralism/Diversity [21–24], Transparency /Accountability [25–27], Recognition [28], and Social and Political impact [29–31] (Table 2). These categories have helped explore how solidarity is being organised, what the barriers are and how they are overcome, as well as the social and political impact derived from it.

**Table 2:** Key dimensions of solidarity analysed in the study

Dimensions	Description
Democracy	The participation of all organization members in governance and decision-making processes is considered as organizational democracy [16, 32]. Democracy is a relevant variable because it influences economic development and social improvements. Democracy protects the rights and freedoms of citizens [19] and promotes inclusive institutions [17]. Thus, citizens can express their voice [18], making their organizations, governments and States more successful.
Pluralism/ Diversity	In terms of the members making up an organization, cultural, ideological, religious and other identity diversities are considered as a competitive advantage [22]. Diversity, together with democratic decision-making processes, enriches the organization, accumulating more social capital and consequently, more effective and successful actions [23]. Thus, some scholars [21, 33] have noted that plurality should be recognised and promoted.
Transparency/ Accountability	Transparency in NGOs has acquired a prominent role in recent years, especially after the economic crisis and specifically for NGOs which are working to reduce inequalities and respond to social needs [25, 34]. These entities are mainly financed through donations from individuals [27, 35, 36], and society is interested in how the funding is used and the actions that these organizations carry out [26, 37, 38]. So, transparency acts as a mechanism of institutional legitimacy, increasing the donors´ trust and thus the ability to continue doing their work.
Recognition	The social recognition of an NGO, such as having received an award, allows to have even greater social visibility and provides powerful incentives to continue their work [28], enabling them to generate a remarkable political impact.
Social and political impact	In the case of NGOs, showing their achievements is the first step to providing organizations´ legitimacy, resulting in performance legitimacy [30]. Social organizations and initiatives are increasingly required to demonstrate that they are improving the lives of the vulnerable groups they serve. Accordingly, social impact is understood as “the social improvements achieved as a consequence of implementing a particular project or action” [31] and “political impact as the institutional repercussions of this project or action”.
Scalability	To address the spatial dimension, we will look at the scope of the organization analysed, as well as the extent of the solidarity action. The relationship between success (in terms of social improvement of peoples’ lives) and scalability should be addressed.

Source: Elaborated by the authors based on the references

## Results

REDER emerged as a civil movement to address the restrictive measures applied after the approval of the RDL 16/2012 and as a solidarity initiative to defend the right to healthcare of groups in irregular situations. Moreover, the philosophy of the network is not only to aid excluded people but also to identify cases of human rights violations in order to report the situation and influence at a political level. In this sense, the case registry comprises a core element of the network, where the affiliated organisations report cases of exclusion.

Prior to the creation of REDER in 2014, Doctors of the World launched a message to social and healthcare professionals and the administration and management staff of the Spanish National Health System, encouraging them to join the conscientious objection movement against the application of the RDL. They also encouraged all citizens to support mobilisations against the RDL led by many social organisations. The conscientious objection movement was a first step for REDER, and the involvement of people who would express their opinion and support the movement was fostered from the beginning.

*“Many awareness-raising campaigns have taken place since the implementation of the RDL, publicly demonstrating the incoherence and injustice of making migrants scapegoats for the austerity policies that have cut back social spending and rights. There are many examples, such as #NadieDesechado, #YoElijoSerHumano or #5MentirasQueDuelen. They intend to boost original and imaginative actions and make citizens identify with people excluded from healthcare by putting themselves in their shoes”.* ([39], CSO4).

The core group of REDER is formed by the Spanish Society for Family and Community Medicine (Sociedad Española de Medicina de Familia y Comunitaria, SEMFYC), Doctors of the World, Observatorio del Derecho Universal a la Salud de la Comunitat Valenciana (Observatory on the Universal Right to Healthcare of the Valencian Community, ODUSALUD), and Andalucía Acoge (Andalusia Welcomes), but the network is open to all who share its objectives. Currently, REDER is formed by 300 organisations and individuals, which financially support the network.

Two core elements comprising the governance of the network are the case registry and the foundational declaration (the “Manifiesto”); both are basic tools for the good management and reporting of cases of exclusion.

REDER activities include periodical meetings to coordinate efforts and guarantee that the information included in the case registry is of good quality.

On deeper level, the activities of the network members are focused on the following objectives:

- Objective 1: The identification of the negative consequences of the adopted RDL 16/2012 measures in the healthcare system.
- Objective 2: The enhancement of the visibility of discrimination in healthcare access and universal health coverage.
- Objective 3: The stimulation of citizens´ participation in initiatives opposing RDL 16/2012 and favouring the network.
- Objective 4: The dissemination of the actions against the restriction of universal public healthcare access.
- Objective 5: The unification of efforts and creation of synergies to restore universal healthcare coverage and accessibility.

According to the key dimensions of solidarity reported in the literature, REDER seems to be a successful solidarity experience. The following subsections corresponding to each of these dimensions describe the characteristics that demonstrate the effectiveness of the initiative from an organisational point of view, as well as the social and political impact deriving from it.

### Democracy

The two core elements, the case registry and the foundational declaration (the “Manifiesto”), reflect the open nature of the network.

The Manifesto is the first step towards adhering to the network, and it was initially developed by the promoters. It states the networks´ mission, vision and objectives [40], and many groups, movements, organisations and individuals committed to defending universal access to healthcare and denouncing non-compliance have signed the Manifesto [40].

The case registry is also an open option to every organisation or social group member who, beyond adhering to the Manifesto, intends to participate in case reporting and the implementation of activities for public denunciation. Exclusion cases are identified through different member organisations who have already established contact with people affected by RDL 16/2012. Organisations that opt for this mode of participation in the network must comply with the minimum requirements and follow the protocol for case registry. Adhering to these requirements is compulsory in order to guarantee high quality reporting and to avoid low-quality information that could undermine the robustness and credibility of the registry. Members who register must follow a strict protocol validation procedure of identification, filling in the confidentiality requirements, the case questionnaire and the file with the definition of the verification process. Core group members guarantee the correct application of the registry and coordination among all members, and they reserve the right to audit and verify all cases.

*“We need to ensure all cases are properly reported and information is correctly collected, not only to adequately solve the vulnerability, but also to preserve the robustness of the data we report”* [CSO4].

*“We use a rigorous protocol from data collection to its statistical use. We then use this information for public denouncement, contact with the administration, judicial proceedings, etc.”* [CSO3].

### Pluralism/diversity

REDER is a participative movement with great diversity, including stakeholders from different areas such as NGOs-voluntary organisations, scientific societies, observatories, platforms and immigrant organizations. The 300 organisations and individuals integrated in REDER seek to join their voices with all the others calling for the urgent reform of the RDL and the subsequent Royal Decree 1192/2013.

Many healthcare professionals refused to collaborate with this new legislation and chose civil disobedience while continuing to provide health services to those excluded, although they ran the risk of being fined [41]. However, in practice, they faced many constraints; as they could not guarantee the transfer from primary care to hospital care or include pharmaceutical benefits.

*“It has been able to reach more than 480 conscientious objectors in the community of Madrid, and although most of the professionals are primary level practitioners, a wide range of professionals from the secondary care level and administration are also represented.”* [NPS].

The initiative has also been able to increase social awareness and communicate the implications for communities, and it involves a very plural citizenship against social injustice.

### Transparency/ accountability

REDER intends to constitute itself as a transparent network, and it promotes this value through its practices. Adhering to its goal of making discrimination visible and thus leading to citizens´ greater awareness and activation, the REDER network intends to publish all collected data and provide periodic reports to the public in general.

There is a large amount of information about forms, guides and the services provided by REDER. Nearly all the information on REDER is available through its website, including periodic reports (with versions in English). Doctors of the World (as part of the network) is active in publishing periodic briefings on REDER. Awareness-raising campaigns are also part of the information sharing and communication channels used by the network. Amongst others, there are the biannual briefings with updated information on the impact of the RDL and REDER.

*“Given the lack of official data on the impact of the measures contained in the RDL, we intended to document and to disclose several reports and documents that record the effects on the health and life of thousands of excluded people. These reports present important elements in order to claim for fundamental rights restoration and protest the RDL’s negative effects”*. [CSO3, NPS].

In terms of financing, REDER is being supported by the organisations that form a part of the network and by a contribution from the Open Society Foundation. Regarding the main coordinating organisations, Doctors of the World is financed by membership contributions, grants for projects, and volunteer work. SEMFYC is a scientific society comprised of family physicians financed by membership fees and a broad number of scientific and dissemination activities. Awareness of the increasing mistrust in society regarding the governance and funding of every organisation or NGO means that core group members care about making sure that everyone has information about the network at their disposal. The open character of the network also attempts to reinforce this point.

The decision-making process is also characterised by its transparency and volunteer involvement to a large extent, not only within the network itself, but also with respect to Doctors of the World. This exercise of transparency and accountability to all volunteers is part of the philosophy of keeping them at the centre of management and decision-making, thus avoiding any conflicts of interest.

*“Participation, not only in concrete initiatives and fieldwork, but also in fields of decision-making is an added value for the organisations and the network as a whole”.* [CSO5].

### Recognition

Although interviewees consider the movement as having credibility and respect, *“it is neither well known by most citizens, nor the social media. This is an obstacle in achieving social involvement and protection for excluded immigrants”.* [CSO1, CSO2].

However, close collaboration and recognition from public administrations with organisations involved in REDER have achieved some progress in the field of political claims and advocacy for citizens´ fundamental healthcare rights.

Due to this acknowledgement and recognised working experience in undocumented immigrant population groups, direct negotiation with those responsible for autonomous health services has been conducted in some autonomous communities, which has contributed to the recognition of the vulnerabilities and revocation of healthcare access barriers for the undocumented immigrant population.

*“We are considered as a reference in the field of assisting and addressing the problems of the most vulnerable groups of people. Thanks to this recognition, our claims and suggestions are being considered by the administration and especially, the Ombudsman”.* [CSO2, CSO4].

Recognition of the network has also promoted the involvement of more professionals in the fight for universal healthcare and the refusal to collaborate on the violation of fundamental rights.

*“Many professionals took the option to attend people excluded by the RDL. And there are many other such cases we are unaware of”*. [CSO4, NPS].

### Health and social impact

The main purpose of the network has been the restoration of the universality of healthcare rights from a policy point of view, and in this respect, the activities of the network have been largely directed towards universal healthcare rights. However, intermediate achievements have been shown to impact health and social aspects, including the number of cases to which it has been providing care, as well as support individual and social groups.

1. *Case registration and problem solving*

Since the creation of REDER, the network’s organisations have provided data from all over Spain and they have recorded more than 4,755 cases of individuals whose human right to healthcare has been violated. An overview of the most affected groups of people and health issues is presented in Table 3. Although the law recognises assistance to these groups of people, in practice, they have been wrongly denied healthcare. To these groups, the number of regular immigrant families with residence permits that were excluded and affected by the RDL should be added.

**Table 3:** Figures of exclusion based on registered cases (2014–2018)

Excluded group, disease or cause of exclusion	Number of registered cases
Pregnant women	194
Minors	322
Elderly people who have been refused health card	78
Asylum seekers	45
Improprer billing or healthcare refusal at emergency units	443
Cases of cancer	68
Cases of hypertension	92
Cases of HIV	45
Cases of cardiovascular disease	88
Cases of diabetes	116
Cases of serious mental health	68

Source: Elaborated by authors based on references [36]

These numbers are only representative of the people who have been able to contact one of the social organisations or collectives that are part of REDER to receive support, advice or medical care thanks to engaged professionals. The network has been able to solve over 90% of these cases either by providing healthcare services or administrative support. Despite variability between regions in the responses to claims, most of the reported cases have been solved by providing the needed care, solving administrative issues or providing guidance and information.

*“There is a lack of homogeneity amongst not only regional regulation, but also healthcare centres or professionals that attend citizens”*. [CSO1, CSO2].

*“In the Basque Country, for example, we had offered a solution to almost all the cases through the public healthcare system”* [CSO4]. *“A similar situation has happened at national level”* [NPS].

*“Most cases correspond to people who have stopped going to healthcare centres or emergency rooms because of fear of being denied care or asked to pay for the assistance. Our principal responsibility was to inform them on their rights as citizens to receive emergency care, and in some cases to restore health through the health professional net, who were available for caring those in most urgent and vulnerable situations”.* [CSO 4].

This is a representative sample in terms of portraying the suffering and impact on human lives caused by the RDL.

*“We have a wide casuistic of people that come to the centres, from pregnant women, chronic patients who have been denied their right to assistance, to mothers with minors who do not know their rights to healthcare”*. [CSO1, CSO5]. *“( … ) and we are aware that these numbers are just the tip of the iceberg”.* [CSO3, CSO4]

Furthermore, another side effect of the RDL that is especially serious and cannot be overlooked is the loss of opportunities to identify undocumented migrant women at medical consultations or any other setting in the health system who are victims of gender violence.

Another main aim of the network is to inform and provide excluded people with necessary information to demand their rights on their own.

*“It is not worth to help them at a medical consultation if you do not empower them. This can only be a short-term solution”.* [CSO4].

*“We are not only trying to solve individual cases, but to change the current health system that excludes vulnerable population groups”.* [CSO4, NPS].

2) *Social awareness towards activation*

The adoption of the RDL has had a great impact on the culture of the population and communities. People have not been exercising their rights due to mistrust and a lack of awareness of their means to exercise their fundamental right to healthcare.

Confusion about the terms of the restrictions was seen among the targeted population, but also doctors and other stakeholders in the health system, many of whom sometimes give the wrong information or hinder access to healthcare services for people who are eligible for assistance without any restrictions.

*“A culture of “no rights” has been installed; people are afraid due to misinformation and this is resulting in immobility. REDER and our organisation aim to reverse the situation through people’s empowerment”.* [CSO4, CSO5].

*“People are already being denied health care due to professional misinformation of the legislation in force”.* [CSO5, NPS].

Furthermore, the implementation of the RDL has indirectly affected the immigrant population with regularised status, either as victims of the deterrent and fear-inducing effects of administrative actions (such as the placement of posters in hospitals and healthcare centres or the lack of adequate information on the restrictions instituted by the reform) or the absence of other types of information campaigns by public administrations.

3) *Establishment of support groups*

In view of the consequences that misinformation and lack of awareness brought about, educating groups of excluded people, as well as other civil initiatives, have played a key role in increasing social awareness and made people aware of their rights, informing them of the process to access these services and increasing their capacity to claim and fight for respect of their fundamental human rights.

An important role is played by the information and support groups that have been formed by various social organisations and citizen platforms. In contrast to the tremendous lack of information that characterised the setting up of the new regulation, which meant that many immigrant people did not even dare to go to emergency units, these groups go to great extents to explain the options for receiving attention.

Support groups also accompany excluded people to healthcare centres and hospitals to mediate and demand that they be treated, and they even offer legal support in order to exercise the rights of excluded people when possible, which shows the social compassion and solidarity network that REDER has been able to build.

The network also prepares and trains its own staff regarding issues that affect all those people who were expelled from the National Public Health System [38]. The beginnings of this process was not easy, because there was a great deal of confusion. However, throughout 2013 and 2014 the SIAD team, an information, accompaniment and denunciation service with offices in Madrid City, was consolidated in a more systematic and effective manner. This service has been developed by Doctors of the World within REDER network to provide information, administrative support and to provide public awareness processes in the cases of exclusion due to the legislation.

Confusion, conflicting interpretations of the legislation, and the attitude and reluctance of many professionals at the healthcare centres have made decision-making and solving these cases difficult [39].

In view of this problem, informative workshops are regularly held in hospitals, health centres, universities and associations, both in Madrid and in other cities in the region, with the objective of training and informing social and healthcare professionals mainly on the regulative situation of each region.

*“We also organised workshops at education centres to inform about the injustices that are taking place and explain myths surrounding immigrants and healthcare use. This has led many teachers in the Madrid region to send letters to the ministry asking for action and a stop to exclusion of the immigrant population from healthcare”.* [CSO3].

All these legal changes have created the misunderstanding that there are no rights with respect to healthcare assistance for undocumented immigrants. In REDER’s initiative, other activities are carried out primarily to raise the general populations´ awareness concerning the situation and the human rights violations that the law implies, intending, above all, to dispel myths about immigrants´ misuse of resources.

Regarding social activation, activities have contributed to citizen empowerment in the following aspects:

- Support acting out and claiming fundamental human rights in order to encouraged vulnerable people to demand respect for their rights as citizens.
- Education in terms of the rights they already have recognised by law and procedures to access assistance and claim the right to be attended at healthcare centres.
- Training organisations and professionals that work with immigrants on what the law already recognises and on how to act against human rights violations.
- Empowering people and organisations to claim their fundamental rights, such as universal healthcare access.

### Political impact

In Spain, REDER has been the main network joining individuals and groups in the fight for universal healthcare restoration. Its leadership in social mobilisation has resulted in pressure at the political level to restore universal access to healthcare.

Although it is not possible to directly link the adoption of all these measures to the activism of REDER, it can be stated that the network has been active in the change in language at the political level.

1. *Advocacy and recommendations for change*

The network has been active in boosting change through advocacy and social activation activities against healthcare exclusion and human rights violations after the enactment of the RDL 16/2012. With this in mind, Doctors of the World (as part of the network and within its objectives) recommends that the Spanish Government restores the health model in line with the principle of universal healthcare.

Faced with the discriminatory situation, social and political pressure from several social organisations, as well as other mechanisms at the European level in favour of a universal healthcare system and the protection of human rights, has also been applied.

2) *Partners and achievements at regional level*

Some achievements could be directly attributed to the work done by the network, such as the elimination of legal requirements to obtain health cards or the reduction of minimum time required.

Even if it is not possible to completely attribute the activity of REDER to its success, it can be stated that due to all this activity in defence of universal care, most autonomous communities have adopted measures to give a certain degree of access to excluded people, even if later judicial sentences have revoked them.

The success of the work done to make exclusion cases visible by means of the registry and complaints made to the Ombudsman is remarkable, and the network has been able to gain regional recognition and respect for their claims and ideas. Due to this and social and media pressure, at the regional level, the autonomic administrations have temporarily introduced regional legislation granting healthcare access to undocumented immigrants who have been living in the region for a certain period of time [3].

In general, these are measures aimed at addressing public health issues or cases which may receive some media attention because of their specific relevance. However, the measures taken by the autonomous communities vary, thus causing significant disparities and inequalities.

A paragraph from a REDER report (2018) acknowledges this complexity of the legislation at the regional level: “… measures adopted by each Autonomous Community without any coordination with the state has given rise to a highly fragmented territorial situation, with 17 regional healthcare systems that recognize different degrees of coverage and have different acceptance requirements”.

*“ … just imagine the enormous complexity of navigating this system for an immigrant who changes their residency from one region to another”* [CSO4].

In a context where regional administrations were enacting legislation to change the impact of the RDL, high advocacy puts pressure on political decision-makers. In March 2015, the central government announced that the undocumented immigrant population would recover their right to primary healthcare, but not their healthcare cards. This announcement never resulted in any legislative measures, and there was no rectification of the RDL 16/2012 measures by the government.

In addition to REDER’s activity, other official institutions, such as the various ombudsman offices, have also reported on the situation, and the reports of the RDL were issued by various European and international human rights organisations, such as the European Committee of Social Rights, the Committee for the Elimination of Discrimination against Women, and the United Nations Special Rapporteur on Extreme Poverty, together with other United Nations Special Rapporteur posts [8]. Even though they do not directly work together, all these activist activities respond to the same demand, and internationally made claims have been acknowledged by REDER reports and the network communication channels.

One of the milestones of the work done by the social movement was an agreement signed on September 12th, 2017, where almost all of the political parties in the Parliament committed themselves to the defence of a universal and public healthcare system. The first commitment was the presentation of a draft law to guarantee health access to every citizen living in Spain, regardless of their administrative situation. The initiative was fostered by REDER.

On June 2018, the recently elected Spanish Government started talks with the regions and the civil society on re-establishing the universality of the Spanish National Health System. The new Minister of Health, summoned the autonomous communities on June 28th, 2018 for an Interterritorial Council, focused on universal coverage.

In September 2018, the Spanish Parliament passed a new universal coverage regulation following a change in the Spanish Government. The new RDL re-established the universality of the Spanish National Health System, after the Ministry of Health opened a process of dialogue with the regions and the civil society. The newly approved RDL 7/2018 on universal access to the National Health System set out to put an end to more than 6 years of exclusion from health care. However, almost 3 months after its approval and more than 1 month after it was ratified by the Congress, it is still not being applied in practice [42].

A sustained and coordinated fight by the Spanish civil society, supported by the Center of Economic and Social Rights (CESR) and other international actors, obtained a firm promise from the new government to repeal the decree [43].

*“Beyond political interests and discrepancies amongst parties, consensus between almost all the opposition political parties has been reached on a non-legal proposal for the derogation of the decision/law 16/2012. The network has been able to unify most representatives in Parliament against the RDL”.* [CSO1, CSO2, CSO3, CSO4].

All in all, from its creation to the present, the network has been able to achieve many of its objectives. Table 4 shows key facts regarding the network, both in terms of the successful results of its human rights violation registry and as a social movement for political involvement.

**Table 4:** REDER’s impact from January 2014 to September 2018

REDER. Rights violation registry	REDER. Social movement and political incidence
Registration of healthcare rights violation cases, with 4,755 cases of excluded people, of which 55% were in an irregular situation and 19% have EU citizenship.	Creation of information and support groups promoted by various social organizations and citizen platforms.
Identification of cases where the RDL recognized healthcare assistance (such as cases of pregnant women, minors, impropriate billing from emergency units).	Condemnation of the RDL by various European and international human rights organizations.
A wide casuistic of documented cases in 14 autonomous communities and Melilla.	Regional regulations to guarantee assistance for excluded populations.
More than 90% of cases solved (either by healthcare assistance or administrative support).	Pact signed by all the opposition political parties to commit themselves to defend a public, universal and quality NHS on 12th September, 2017.
Implication of more than 2,200 professionals as conscientious objectors at the national level, mainly general practitioners.	Social cohesion and generation of empathy for the defense of fundamental healthcare rights for the most vulnerable populations.

Source: Elaborated by the authors based on references and interviews [10]

### Scalability

The core elements, such as the case registry and the Manifesto, have been scaled to national (Spain) level. However, due to the variety of legislation at the regional (autonomous communities) level, other kinds of actions are focused at a local level. In this sense, many specific actions in the framework of the social movement have been regional or local in scope, adapting them to the context and requirements of each place.

Of the many efforts made, it is worth highlighting the work done at the headquarters in Madrid where the SIAD team was launched in 2012 within the context of the healthcare exclusion that caused the implementation of the RDL 16/2012. They inform people seeking solutions to their health situations, accompany them if necessary to solve their problems at health centres or hospitals, and report cases of people denied health care even when they have the right to it according to the RDL. At the same time, in the Basque Country, el Cassin (located in the city of Bilbao) is an example of this kind of support and information centre.

The number of people assisted in both the examples of Madrid and the Basque Country has been increasing since the SIAD’s team beginnings. All the cases in the two places are recorded in two databases, in addition to completing another file with the testimonies of people who visit their facilities. The increase involved some difficulties in the initial stages, due to the lack of time and resources to attend to all of them in the best possible way.

*“Although in the 2000s´, we saw how the number of people that came to the centre decreased because of changes in legislation, since the RDL, we have to increase the number of days and hours we open the centre, after having cut office hours to two days a week*” [CSO5].

These initiatives show how similar actions can be taken in different contexts, prior adaptations and consideration of the local situation with respect to legal, cultural and organisational aspects, amongst other variables.

Furthermore, the lessons learned and the main actions carried out are scalable to other countries, where rights to healthcare access are being reduced. Initiatives such as the European Network to reduce vulnerabilities in health can help in scalability efforts [44].

The analysis of the different characteristics and actions of REDER, and their linking to specific solidarity dimensions, shows how the network has been able to fulfil its objectives. Table 5 reflects how different aspects of each of the dimensions contributed to the objectives of REDER described in the paper.

**Table 5:** Relation between characteristics and goal achievements of REDER

Dimension	Achieved goals
Democracy	O3, O5
Pluralism/Diversity	O3, O4, O5
Transparency/Accountability	O1, O2,
Recognition	O1, O2, O5
Social impact	O1, O2, O3, O4, O5
Political impact	O4, O5
Scalability	–

O1: Objective 1; O2: Objective 2; O3: Objective 3; O4: Objective 4; O5: Objective 5

Source: Elaborated by authors

Both the identification and dissemination of the negative consequences of the enactment of the RDL (O1, O2) have been achieved thanks to the transparency of all of the information managed by the network and the effort to complete a registry of exclusion cases.

The open nature and the diversity of individuals and organisations participating in REDER is also an example of how it has stimulated and achieved citizen’s involvement in activities opposing the RDL (O3). The unification of actions, the diversity of stakeholders involved, and social activation have created synergies to create pressure in the political sphere.

## Discussion

This study provides insights into the effectiveness of the REDER solidarity initiative for the protection of the universal right to healthcare, while analysing its characteristics according to key dimensions identified in the literature on solidarity. The study provides data on the origins, organisation, membership, and activities of REDER in order to identify key elements that could have led to its success as a social solidarity movement.

Previous research has shown how other solidarity initiatives in different countries in Europe have performed regarding each dimension of solidarity and how they achieved social impact [45], reinforcing the relevance of those aspects for success.

Once more, this research shows that REDER’s organisational aspects, its nature and its activities have been effective with respect to its main goals of advocacy and reversal of measures that eliminate the universality of health care.

The organisation of a group, as well as the social context in which a group operates, is an important factor for activating and engaging citizens in solidarity initiatives. Prior research has identified superordinate goals, support for intergroup contact, and equal group status as important elements in establishing intergroup cooperation and solidarity [45]. In this case, issues regarding the internal organisations of REDER, with a focus on accountability-issues, transparency of the working-process and the funding-system as well as structures for democratic decision making, show how they contribute to its success.

The factors causing poverty and social exclusion have hit immigrants hard, especially illegal immigrants, with significant restrictions of their rights, particularly in relation to healthcare [46]. There are several restrictions beyond the Spanish borders that prevent immigrants from accessing basic healthcare services, including emergency care. These policies explicitly state that undocumented immigrants cannot not seek health services or contain clauses that prevent them from seeking healthcare services. Therefore, being “undocumented” was used as a means of exclusion from vital services [10, 47].

However, as shown by REDER, other studies have also indicated that the use of the healthcare system by immigrants is not higher than that of the local population [48, 49]. Therefore, there does not seem to exist any objective data that would uphold the economic reasons for the system’s sustainability mentioned by the Spanish Government. The implementation of the RDL has not been able to provide any evidence showing that there is risk to the system’s sustainability [5]. Additionally, this situation hinders timely diagnosis and appropriate treatment of infectious conditions, the implementation of preventive activities, and health promotion. Furthermore, hindering primary care access creates a greater number of emergency room visits, which increases costs for the health system [50]. In Spain, the mortality rate in the immigrant community with irregular status increased by 15% during the first 3 years of the implementation of the reform, and a greater use of emergency services was registered [10]. In addition, restricting access to health care for undocumented immigrants in 2012 considerably reduced the probability of this group visiting a specialist doctor and the probability of them making a scheduled hospital visit [9].

Beyond this, at the time of the implementation of RDL 16/2012, the government announced alternative health care plans for undocumented immigrants, which with time have proven to be unaffordable and even more expensive than existing private insurance plans in Spain [51].

On the other hand, other studies have also identified that a perceived fear of deportation and harassment from the authorities correlated with the lack of access to health services. Immigrants perceived these policies as a threat not only to themselves but also to their families and as sources of criminalisation [52].

To address these restrictions and injustices, several regional governments have adopted some measures to allow certain healthcare access to excluded people. Cimas et al. (2016) have compared regional policies regarding entitlement to healthcare for undocumented migrants after enactment of the RDL in the 17 autonomous communities by performing an exhaustive review of the health policy regulations published after the enactment of the RDL. They concluded that “from a health policy point of view, the unequal implementation of the RDL 16/2012 in Spain is a paradigmatic example of the complexity of national regulation in key issues in decentralised health systems. Regional policies have diminished the intended effect of RDL 16/2012, but there are huge differences in healthcare coverage for undocumented migrants among Spanish regions”.

For the moment, the RDL 7/2018 has been enacted to put an end to exclusion in healthcare. However, in practice there are loopholes that make it possible to carry out the total application of measures to restore the universality of healthcare. REDER continues to conduct its own activities and social mobilisation initiatives, demanding for the integration of the application of the RDL throughout the country to guarantee equal healthcare access to all persons.

In the context of restrictions and human rights violations, solidarity initiatives such as REDER have proven effective in reducing the negative impact and achieving social cohesion, due to their very nature. They has also shown their power in boosting a real change at the policy level. The characteristics and aspects examined seem to contribute to the success of REDER.

Results from the SOLIDUS European Project found common shared drivers in the more than 90 solidarity initiatives analysed around Europe. Democracy, transparency, plurality, social and political impact, recognition, scalability and dialogic interactions were among the common elements shared by all of these initiatives to guarantee solidarity actions with social impact. This study, as part of this project, is an example of how different dimensions contributed to the achievement of the first line goals stated in the networks´ statements.

The study has some limitations that could be addressed in future studies. On the one hand, it has not been possible to interview patients or directly affected undocumented people. This restriction limits the analysis of the network with respect to the results on the activation and empowerment of people in defending their own rights. The incorporation of these kind of profiles in the interviews could be enlightening, allowing us to reflect on the impact of the network from a beneficiary perspective, understanding excluded people as “beneficiaries”.

On the other hand, in a fast-moving context the opinion of interviewees reflects only the period before the enactment of the last RDL 7/2018. The situation and activities carried out after this event are reflected in the paper only through the information collected in public reports and documents.

## Conclusions

REDER, a social solidarity movement, has been shown to be effective in leading the defence of universal healthcare rights. The RDL 16/2012 aimed to restrict undocumented immigrants from accessing healthcare. Furthermore, this immigration policy increased the fear of detention and the lack of knowledge about the health care system, which limited undocumented immigrants’ ability to effectively access health services and affected health outcomes.

Although this research cannot conclude that there is a direct causal link between REDER and a reversal of the law, it can be stated that REDER’s leadership in coordinating the fight of different agents and organisations in Spain against healthcare exclusion has influenced changes at policy level.

Despite context particularities, REDER shows how the main actions promoted by the network may be implemented in other settings facing similar limitations to healthcare access and promote policy level reforms to address injustice and promote solidarity.

## Additional file

Additional file 1: Appendix I Interview guideline. (DOCX 16 kb)

## Abbreviations

NGO: Non-Governmental Organisation
NPS: National Public Seminar
RDL: Real Decreto Ley- Royal Decree Law
REDER: Red de Denuncia y Resistencia al RDL 16/2012- The Network for Denouncing and Resisting the Royal Decree-Law 16/2012
