# Supplementary material for: Solidarity against healthcare access restrictions on undocumented immigrants in Spain: the REDER case study
Source: Int J Equity Health. 2019 Jun 6;18:82. doi: 10.1186/s12939-019-0971-9 (PMC6554933; doi:10.1186/s12939-019-0971-9)
Supplement: Supplementary file 1 — Appendix I Interview guideline. (DOCX 16 kb) [file 12939_2019_971_MOESM1_ESM.docx]

**Additional file 1: Appendix I**

**Interview guideline**

**Characteristics and nature of the organizations**

**Which type of organization is (name of the organization to which the participant belongs)?**

1. Which is its mission, values and main objectives?
2. Which activities does it work on?
3. Which was the context in which the organization was created?
4. Who were the promoters? Which profile do they have?
5. How is the target to which the organization directed its actions?
6. How do you organize the decision-making process? Who take part in the process (volunteers, technical professionals, etc.)?
7. How does this process benefit the organization, if so?
8. How the organization is funded as most?
9. Which are other organizations and allies with whom it collaborates, if so?

**Social, health and economic impact of organizations activities on users**

1. When and why do you decided to take part on the organization?
2. How does your work and participation in its activities change your mind, if so?
3. Is there any economic reason/motivation for your affiliation?
4. Describe a situation in which users have change de development of activities or the direction of the organization.
5. Which are the main achievements in terms of health and social support to excluded subgroups of population?
6. Which are the main achievements in relation to the empowerment of excluded subgroups of population?
7. Which were facilitators for the success? Which the barriers?
8. How does social media contribute to it, if so?
9. Which is your opinion on the impact that the organization has had through its activity on public administrations, public policies?
10. How do you collaborate with public administration, if so?

**Future collaborations and measures to respond restrictions**

1. Do public institutions and administration recognized the activities done by the organization?
2. Which type of collaborations could be effective between third sector and public administration?
3. Which kind of legal barriers could be for collaboration?
4. How could it be promoted a more egalitarian and supportive society between third sector and public sector organizations?
5. Which strategies or proposals do you have for the future?
